# The long-term effects of COVID-19 on oxygen carrying capacity in adults at the University of Limpopo, South Africa

**DOI:** 10.3389/fmed.2025.1636141

**Published:** 2025-11-24

**Authors:** Kidibone Mkhabela, Marlise Van Staden, Yvette Yolanda Chetty

**Affiliations:** Department of Physiology and Environmental Health, University of Limpopo, Sovenga, South Africa

**Keywords:** hemoglobin, haematocrit, COVID-19, South African, long-term effects

## Abstract

**Background:**

Long-COVID is a condition characterized by persistent symptoms that last past the acute phase of COVID-19. The symptoms manifest from COVID-19 infection, and include shortness of breath, fatigue, and headaches. These symptoms could be caused by hypoxia and hypoxemia which may result from low haematocrit and hemoglobin. The SARS-CoV-2 virus causes destruction to the heme group of hemoglobin leading to haemolysis, which may lead to disrupted oxygen-carrying capacity.

**Aim:**

The aim of the study was to investigate the long-term effects of COVID-19 on hemoglobin (Hb) and haematocrit (Hct) in adults.

**Methodology:**

This was a case-control cross-sectional study that included a COVID-19 non-naive group (*n* = 28) and a COVID-19 naïve group (*n* = 196). Questionnaires were administered to participants, and blood levels of Hb and Hct were measured. Furthermore, a food frequency questionnaire (FFQ) was administered to determine the daily intake of iron, folic acid, and vitamin B_12_ to observe if the diet might have had an influence on Hb and Hct.

**Results:**

The results show a trend for a low Hct is common in females who had a history of a positive COVID-19 test, and the trend for a normal Hb is prominent across the study population. Results also show a statistically significant (*p* = 0.001; 95% CI −1.064 to 9.295) intake of vitamin B_12_ in COVID-19 non-naive males, who also showed to have the highest levels of Hb and Hct (mean of 45.86 g/dl and 15.57% respectively) as compared to other groups.

**Conclusion:**

COVID-19 patients in this study experienced persistent symptoms, but most of the participants had no symptoms at the time the study was conducted. Diet might have had an impact on Hb and Hct in the COVID-19 non-naive male population, as it was observed that they had the highest nutrient consumption and the highest concentrations.

## 1 Introduction

Coronavirus Disease 2019 (COVID-19) can be defined as an infectious disease that is caused by severe acute respiratory syndrome coronavirus-2 (SARS-CoV-2) (1). The SARS-CoV-2 targets certain cell receptors to infect its host (2). The cellular receptors (APF2) expressed in the sensory of the carotid body might be directly affected by the virus, causing dysfunction, and reducing the sensitivity of glomus cells to oxygen deficiency (2, 3). Thereby, leading to hypoxia and hypoxaemia. The other mechanism is SARS-CoV-2′s direct effect on the heme group of hemoglobin by binding to CD147 which leads to an interaction between the erythrocytes and the virus (4). This interaction might lead to the cell lysis of the erythrocytes, possibly resulting in the insufficient availability of hemoglobin to transport oxygen (6). The virus could also affect erythrocytes by having ORF8 protein and surface glycoprotein of the virus to bind to porphyrin, while ORF1ab, ORF10, and ORF3a proteins can interact with the 1-β chain of hemoglobin resulting in the dissociation of iron and porphyrin molecules (3–5). Iron forms a vital part of the heme group by binding with the porphyrin molecules, as such a hemoglobin molecule that lack iron atoms is dysfunctional and leads to impaired gas-transporting function (6).

Disturbed iron metabolism is another physiological process that could play a role in the prognosis of hypoxia and hypoxaemia (34). The infection of SARS-CoV-2 induces a hyperinflammatory response, known as a cytokine storm, which involves the overexpression of proinflammatory cytokines, such as interleukin (IL)-6 and IL-10 (7). These ILs can aggravate the acute respiratory distress syndrome (ARDS) and lead to fatal tissue damage (7, 8). Hyperferritinaemia and altered iron homeostasis are some of the signs of the hyperinflammation found in COVID-19 patients (9). Claise et al. (10) suggested that hyperferritinaemia and decreased serum transferrin levels reflect a state of intracellular iron overload. Hyperinflammation leads to the release of microphages that results in the increase in ferritin, which may also increase due to damaged hepatic cells (11). Serum ferritin loses part of the iron content when released and produces extremely high levels of free iron (6). The high levels of free iron leads to inflammation and toxicity of the alveolar macrophages leading to oxidative damages of the lung tissue (12). The damaged lungs will result in impaired gas-exchange, and hemoglobin loses its ability to bind oxygen, causing oxygen deficiency (11). The body will attempt to recompensate by increasing hemoglobin synthesis, thus the high levels of Hb found in COVID-19 patients (6).

The effects of the infections range from mild to severe and have been found to last past the decrease of the viral load (13). Long-COVID is a condition characterized by persistent symptoms that last past the acute phase of COVID-19 (14). Many recovered COVID-19 patients suffer from debilitating symptoms, such as weakness, headaches, fatigue, dyspnoea, cognitive impairment and smell and taste disturbances (15). Studies regarding Long-COVID have been given little attention as resources were aimed at combating the spread of the virus, but the Long-COVID is still affecting many individuals, therefore this study aimed to explore the effects of Long-COVID on hemoglobin and haematocrit, to observe whether individuals who were diagnosed with COVID-19 might still have impaired oxygen carrying capacity. COVID-19 was a pandemic that affected the whole world, but studies conducted in the small townships to observe the effects in those areas are scarce.

## 2 Methodology and materials

### 2.1 Sampling procedure

**Figure 1:**
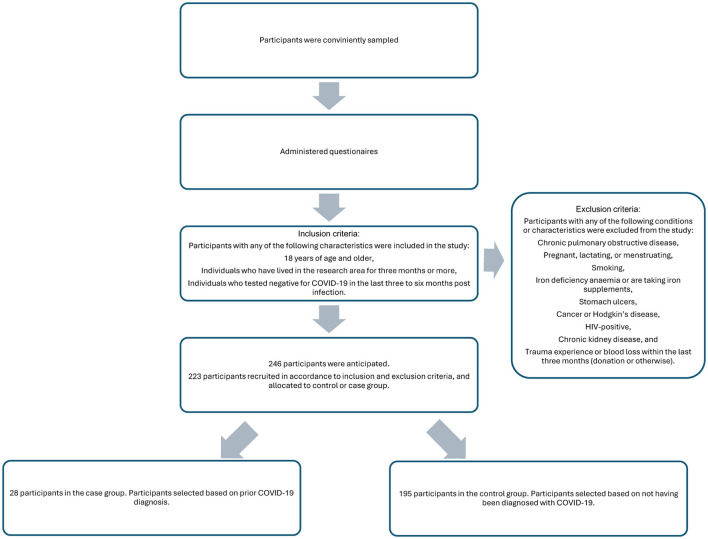
Sampling procedure.

The study was a case-control cross-sectional study, and it utilized quantitative and qualitative methods to acquire data. The data included a range of information such as demographic data, procedures, and diagnoses. The data was collected at the University of Limpopo from 223 registered university students and staff (Figure 1), in Mankweng Township, Limpopo Province, South Africa. The participants were recruited using convenience sampling. Ethical approval was granted by the Turfloop Research Ethics Committee (TREC), project number TREC/370/2022:PG.

The participants were selected in accordance with the inclusion and exclusion criteria (Figure 1). These criteria ensured that the participants had no confounding factors that developed before the infection and could have played a role in increasing the susceptibility for infection, as the study was investing the long-term effects of the COVID-19 on interfering with oxygen-carrying capacity by affecting hemoglobin concentration and haematocrit. The selected participants were given a dietary assessment to determine their dietary intake of iron, folic acid, and vitamin B_12_. A questionnaire was used to eliminate malnourishment as a cause of low Hb and Hct if found in the participants.

### 2.2 Hemoglobin and haematocrit measurements

As described by Billet (16) and Rovó et al. (17), Hb concentration and Hct were measured using the standardized finger-prick method using a FORA^®^ 6 Connect portable device, with the FORA^®^ six test strips that produced the readings for blood Hb and Hct. This method is more cost and time-effective compared to lab-based methods. Results were obtained and presented to participants within seconds. The study lacked research funding, therefore the use of this method was more affordable. However, it limits the number of hematological parameters that could be analyzed. The World Health Organization (35) classified the Hb concentration and Hct into lower-than-normal, normal, and higher-than-normal for males and females. These classifications were adopted to classify the participants accordingly.

### 2.3 Dietary intake

A food frequency questionnaire (FFQ) was administered in accordance with the dietary intake guidelines by the South African Medical Research Council (SAMRC). This questionnaire was found to be the most reliable to acquire daily dietary information as participants had to recall their diet of the past 30 days beginning with the day the questionnaire was administered. Its reliability is based on the participant recalling foods and beverages consumed on a regular basis, and not something consumed the day or night before which might vary according to the consumer's preference. The data was analyzed with FoodFinder software, which were then compared with the recommended daily allowance (RDA).

### 2.4 Statistical analysis

Statistical analysis of the collected data was conducted using the IBM SPSS statistics software package (version 29.0). The data from the FFQ were captured on the South African Medical Research Council's (SAMRC) SAFOODS FoodFinder software and further analyzed with IBM SPSS. Descriptive statistics were produced for all the variables to provide frequencies, means and standard deviations to describe and characterize the samples. The normality of the variables was evaluated by the Shapiro-Wilk test. The student *t*-test was used to test for statistically significance of Hb and Hct in males and females in the COVID-19 non-naive and COVID-19 naïve groups. The Chi-square and Pearson correlation factor (*r*) was used to test for linear correlation between the iron, folic acid, and vitamin B_12_ consumed on a regular basis and Hb and Hct of the participants. A *p*-value of *p* ≤ 0.05 was used as the statistical significance for all tests.

## 3 Results

### 3.1 Characteristics of the population

Figure 3 shows the age distribution across the study population, where majority of the participants were in the 18–25 age range. The age range with the least number of participants is the 42–48 age range.

Table 1 depicts the demographic characteristics of the participants. The data is presented as mean ± SD or *n* (%) and *p*-value, where *p* < 0.005 is statistically significant. The results showed that age was statistically significant (*p* = 0.028) between the cohorts, whereby the total mean ± SD was 21.1 ± 4.3 and for COVID-19 non-naive group was 22.8 ± 7.2 and COVID-19 naïve group was 20.9 ± 3.7. The other characteristics showed no statistical significance, while others had a computational error as some of the variables where constant or were categorical.

**Table 1:** Characteristics of the participants.

**Characteristics**	**Total**	**COVID-19 non-naive**	**COVID-19 naïve**	***p* Value**
	***n*** = **223**	***n*** = **28**	***n*** = **195**	
Age	21.1 ± 4.3	22.8 ± 7.2	20.9 ± 3.7	**0.028**
Sex (females)	135 (60.5%)	21 (75.0%)	114 (58.5%)	^a^
Sex (males)	88 (39. 5)	7 (25.0%)	81 (41.5%)	^a^
Hb ()	12.8 ± 2.7	12.4 ± 2.9	12.9 ± 2.6	0.329
Hct (%)	37.9 ± 7.8	36.4 ± 8.4	38.1 ± 7.7	0.290

Data presented as mean ± SD (standard deviation) or n (%) and p-value.

^a^Computation error as one of the variables are constant. The values in bold show where statistical significance.

Table 2 indicates the frequency of variables in the classifications of the participants. The participants' Hb and Hct were classified according to WHO classification in Table 3, whereby any values below those ranges are considered to be low, and any values above those ranges are considered to be high. It was observed that 85.7% of the COVID-19 non-naive males and 74.1% of the COVID-19 naïve males had normal Hct, while 66.7% of the COVID-19 naïve females and 61.3% COVID-19 females had low Hct. Therefore, showing that Hct was insignificantly higher in the COVID-19 non-naive naïve than the COVID-19 non-naive group in both males and females. It was observed that 100% of the COVID-19 non-naive males and 82.7% of the COVID-19 naïve males had normal Hb concentrations, while 66.7% of the COVID-19 non-naive females and 65.8% of the COVID-19 naïve females had normal Hb concentrations. There was no statistically significant association observed between the two groups with regard to Hct and Hb concentration.

**Table 2:** Trends of Hct and Hb of COVID-19 non-naive and COVID-19 naive group based on sex.

**Variables**	**Total population (*N* = 223)**	**COVID-19 non-naive group (*n* = 28)**	**COVID-19 naïve group (*n* = 195)**	***p*-Value**
Males (*n* (%))	88 (39)	7 (25.0)	81 (41.5)	0.050
Females (*n* (%))	135 (61)	21 (75.0)	114 (58.5)	
**Hct (***n* **(%))**				
**Male**				
Normal	66 (75.0)	6 (85.7)	60 (74.1)	0.754
High	3 (3.4)	0 (0.0)	3 (3.7)	
Low	19 (21.6)	1 (14.3)	18 (22.2)	
**Female**				
Normal	43 (32.6)	6 (28.6)	37 (33.3)	0.896
High	7 (5.3)	1 (4.8)	6 (5.4)	
Low	82 (62.1)	14 (66.7)	68 (61.3)	
**Hb (*****n*** **(%))**				
**Male**				
Normal	74 (84.1)	7 (100.0)	67 (82.7)	0.487
High	1 (1.1)	–	1 (1.2)	
Low	13 (14.8)	–	13 (16.0)	
**Female**				
Normal	87 (65.9)	14 (66.7)	73 (65.8)	0.721
High	8 (6.1)	2 (9.5)	6 (5.4)	
Low	37 (28.0)	5 (23.8)	32 (28.8)	

Hct, haematocrit; Hb, Hemoglobin.

**Table 3:** WHO classification.

**Heamatological parameters**	**Males**	**Females**
Haematocrit	40%−54%	36%−48%
Hemoglobin	14–18 g/dl	12–16 g/dl

Adapted from WHO (35).

### 3.2 Association for haematocrit and hemoglobin

Table 4 indicates the mean and standard deviation of Hct and Hb among the male and female participants in the COVID-19 non-naive and COVID-19 naïve groups. None of the groups had a statistically significant association for Hct and Hb.

**Table 4:** Descriptive statistics of Hb and Hct among males and females in the COVID-19 non-naive and COVID-19 naive group.

**Variables**	**COVID-19 non-naive group**	**COVID-19 naïve group**	***p*-Value**	**95% Confidence Interval (CI) of the difference**
	**Mean** ±**SD**	**Mean** ±**SD**		**Lower–Upper**
**Hct**				
Male	45.86 ± 3.436	43.48 ± 5.259	0.301	−1.660 to 6.411
Female	33.29 ± 7.072	34.17 ± 6.770	0.771	−4.095 to 2.324
**Hb**				
Male	15.571 ± 1.163	14.716 ± 1.829	0.268	−0.547 to 2.258
Female	11.319 ± 2.444	11.586 ± 2.302	0.798	−1.361 to 0.828

Hct, haematocrit; Hb, Hemoglobin; SD, standard deviation.

Figure 3 portrays the differences of Hb and Hct levels between the groups and sexes. COVID-19 non-naive males had the highest mean of Hb and Hct (45.86 g/dl and 15.57% respectively), while the COVID-19 non-naive females had the lowest levels.

### 3.3 Association of micro- and macronutrients that could impact oxygen-carrying capacity

Table 5 indicates the descriptive statistics for iron, folic acid, and vitamin B_12_ between the COVID-19 non-naive and COVID-19 naïve group. The male participants in both groups had no statistically significant association for iron. There was a weak, and statistically insignificant association (*p* = 0.055; 95% CI −4.763 to 0.899) between the females in the COVID-19 non-naive and COVID-19 naïve group for iron. Similarly to the association of iron in male participants in both groups, there was no statistically significant association for folic acid in the male participants. The female participants had a weak and statistically insignificant (*p* = 0.066; 95% CI −75.104 to 21.705) association for folic acid. The male participants had a strong statistically significant association (*p* = 0.001; 95% CI −1.064 to 9.295) for vitamin B_12_, while the female participants had no statistically significant association for vitamin B_12_.

**Table 5:** Descriptive statistics of micro- and macronutrients associated with anemia between males and females in the COVID-19 non-naive and COVID-19 naïve groups.

**Variable**	**Groups**		***p*-Value**	**95% Confidence Interval (CI) of the difference**
	**COVID-19 non-naïve Mean (**±**SD)**	**COVID-19 naïve Mean (**±**SD)**		**Lower–Upper**
**Iron**				
Males	6.943 ± 5.768	7.021 ± 6.669	0.895	−5.255 to 5.099
Females	5.514 ± 3.125	7.447 ± 6.405	0.055	−4.763 to 0.899
**Folic acid**				
Males	90.978 ± 78.184	107.036 ± 102.289	0.466	−94.996 to 62.880
Females	94.923 ± 74.874	121.623 ± 107.272	0.066	−75.104 to 21.705
**Vitamin B**_12_				
Males	9.814 ± 12.240	5.699 ± 5.982	0.001^***^	−1.064 to 9.295
Females	4.576 ± 4.294	4.868 ± 4.739	0.546	−2.487 to 1.904

SD, standard deviation.

^***^p < 0.001.

Table 6 shows the trend of how many participants consumed nutrients above or below the RDA adopted from the NIH (Table 7). All participants in the COVID-19 non-naive group consumed less than RDA of iron, and most of the COVID-19 naïve participants (78 males and 110 females) also consumed less than the RDA of iron while a few of them (three males and four females) consumed more than the RDA of iron. In both groups, majority of the females (15 COVID-19 non-naive females and 72 COVID-19 naïve females) consumed more than the RDA of vitamin B_12_ daily. Similarly with the consumption of iron, majority of the participants in both groups consumed less than the RDA of folic acid.

**Table 6:** Number of participants who consumed nutrients below or above the RDA.

**Classification**	**COVID-19 non-naive**			**COVID-19 naïve**		
	**Iron**	**Vitamin B**_12_	**Folic acid**	**Iron**	**Vitamin B**_12_	**Folic acid**
**Males** ***N*** **(%)**						
Below	7 (100.0)	4 (51.1)	6 (85.7)	78 (96.3)	34 (42.0)	67 (82.7)
Above	–	3 (42.9)	1 (14.3)	3 (3.7)	47 (58.0)	14 (17.3)
**Females** ***N*** **(%)**						
Below	22 (100.0)	6 (28.6)	19 (90.5)	110 (96.5)	42 (36.6)	92 (80.7)
Above	–	15 (71.4)	2 (9.5)	4 (3.5)	72 (63.2)	22 (19.3)

**Table 7:** Recommended daily allowance.

**Nutrient**	**RDA Males**	**RDA females**
Iron	18 mg	18 mg
Folic acid	400 μg	400 μg
Vitamin B_12_	2.4 μg	2.4 μg

Adapted from: https://ods.od.nih.gov/HealthInformation/nutrientrecommendations.aspx.

Figure 4 shows the differences of consumed nutrients between the groups and sex. COVID-19 naïve females consumed more iron and folic acid (7.45 mg and 121.62 μg respectively) as compared to others, while the COVID-19 non-naive females consumed the least iron (4.58 mg) as compared to other groups and the COVID-19 non-naive males consumed the least folic acid (90.96 μg), but more vitamin B_12_ (9.81 μg) as compared to others. COVID-19 non-naive females consumed the least vitamin B_12_ (4.87 μg) as compared to other groups.

### 3.4 Pearson correlation factor for micro- and macronutrients between haematocrit and hemoglobin

Table 8 indicates the Pearson correlation factor (*r*) between iron, folic acid, and vitamin B_12_ and Hct and Hb. Folic acid and vitamin B_12_ had a negative linear correlation with Hct and Hb, but none of the variables had a statistically insignificant correlation with Hct and Hb.

**Table 8:** Pearson correlation between nutrients associated with anemia and Hct and Hb.

**Variables**	**Hct**			**Hb**		
	**Pearson Correlation factor (** ***r*** **)**	**Significance (** ***p*** **-Value)**	**95% Confidence Interval (CI) of the difference (Lower–Upper)**	**Pearson Correlation factor (** ***r*** **)**	**Significance (** ***p*** **-Value)**	**95% Confidence Interval (CI) of the difference (Lower–Upper)**
Iron	0.018	0.794	−0.115 to 0.150	0.018	0.793	−0.115 to 0.150
Folic acid	−0.019	0.783	−0.150 to 0.106	−0.014	0.835	−0.146 to 0.118
Vitamin B_12_	−0.027	0.691	−0.159 to 0.106	−0.042	0.539	−0.173 to 0.091

Hct, haematocrit; Hb, hemoglobin.

## 4 Discussion

**Figure 2:**
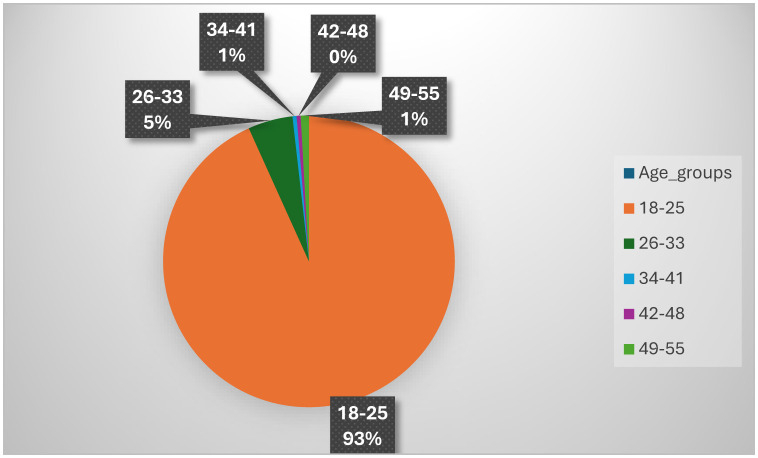
Age distribution.

The aim of the study was to investigate the long-term effects of COVID-19 on hemoglobin and haematocrit in adults. Majority of the participants in this study were within the same age group, 18–25 years old (Figure 2), but a statistically significant value was observed (Table 1) when comparing the age (in years) between the cohorts. The results of the study indicated that the 66% of the COVID-19 non-naive females had and 61.3% of the COVID-19 naïve females had low Hct. This indicates a possible higher prevalence of low Hct in females with prior positive COVID-19 test. The outcome of the results could be due to females genetically predisposed to a lower Hct than males (18, 19), and/or due to the SARS-CoV-2 targeting the CD147 receptors expressed by erythrocytes (20). None of the results observed on the trends of Hct showed any statistical significance. This could be due to participants having had sufficient time to recuperate from the effects of the infection. It can also be that the symptoms differ between individuals and that these individuals just did not have abnormalities in Hb or Hct. It can also mean that the subjects initially had abnormalities in Hb and/or Hct, but that it disappeared over time. Thus, the effects of long-Covid may not be permanent. A study by Pereira-Roche et al. (21) found that in some patients with Long COVID, hematological factors such as hemoglobin levels have returned to normal values once the viral load has decreased, which substantiates the trends observed in this study.

**Figure 3:**
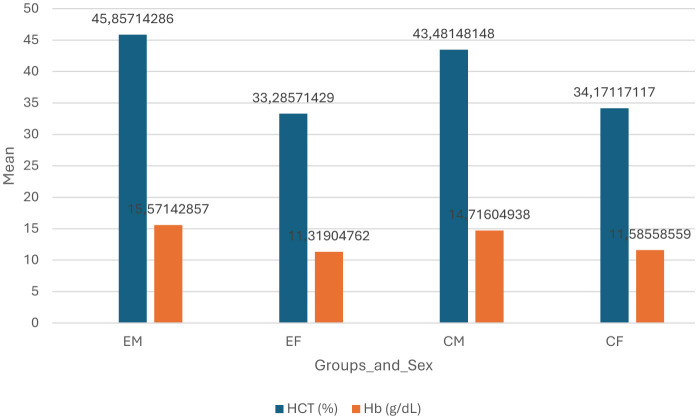
Differences of hemoglobin (Hb) and haematocrit (Hct) in groups and sexes. EM – experimental group (COVID-19 non-naive) males; EF – experimental groups (COVID-19 naïve) females; CM control group (COVID-19 naïve) males. CF, control group (COVID-19 naïve) females; %, percentage; g/dl, grams per deci-liter.

Most males and females (84 and 66%, respectively) had normal hemoglobin levels. This indicate that the participants who had a prior positive diagnosis of COVID-19 have recuperated from the infection and now have normal Hb levels, as Leon-Lopez et al. (15) reported that 80% of COVID-19 patients experience persistent symptoms for a few weeks after the viral load decreases. The trends observed in Figure 3 shows that COVID-19 non-naive males had higher levels of Hb and Hct as compared to the COVID-19 naïve males, whereas the opposite is observed in the females. This suggests that males might have an increased oxygen carrying capacity after recovery than their female counterparts who might have decreased oxygen carrying capacity due to the decreased levels of Hb and Hct. A study by Sonnweber et al. (22) observed that the prevalence of anemia decreased significantly over time in their cohorts. They studied post-COVID-19 recovery patients longitudinally where they conducted tests for anemia and other parameters at 60, 100, 180, and 360 days of recovery. They observed a significant decrease with every assessment, indicating that people do recover from the persistent symptoms and the effects of Long-COVID after a certain duration. Participants of this study were asked about the date of their diagnosis, but none could recall the exact date, only the year, and most were diagnosed in the years 2020 and 2021, when the spread of the virus was still at its strongest. This indicates that over a year has passed for most of the participants as this study was conducted in 2023, therefore full recovery is expected based on the recovery timeline in the literature, substantiating the trend observed in this study.

**Figure 4:**
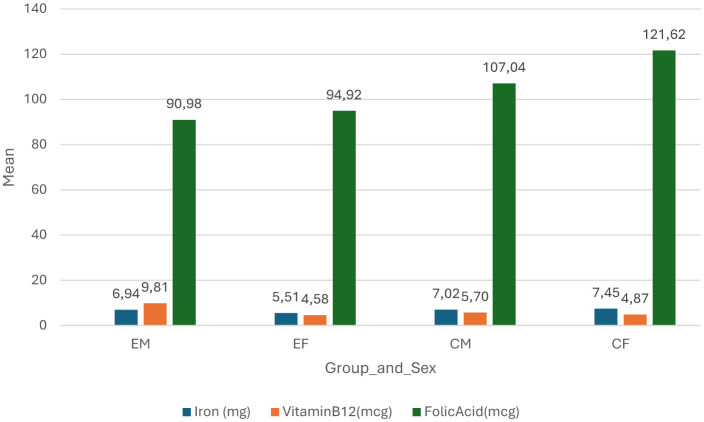
Difference of nutrients between groups and sex. EM, experimental group (COVID-19 non-naive) males; EF, experimental group (COVID-19 non-naive) females; CM, control group (COVID-19 naïve) males; CF, control group (COVID-19 naïve) females; mg, milligrams; mcg, micrograms.

The results also indicate that majority of the participants consumed below the recommended daily allowance (RDA) for iron, folic acid, and vitamin B_12_, but there was statistically significant difference in males who had COVID-19 consumed higher RDA than males who were COVID-19 naïve. However, the observed trend in Figure 4 indicated that COVID-19 naïve females had the highest consumption of the iron and folic acid, while COVID-19 non-naive males had the highest consumption of vitamin B_12_. This finding corroborates the statistically significant value (*p* = 0.001) observed in Table 4. The COVID-19 naïve males consumed more iron and folic acid compared to the COVID-19 non-naive males who had the highest consumption of vitamin B_12_, but the lowest of iron and folic acid. These trends contradict the observations in the Figure 3, as the COVID-19 naïve males had lower Hb and Hct levels as compared to the COVID-19 non-naive group. On the other hand, COVID-19 naïve females consumed more of all three nutrients as compared to COVID-19 non-naive females, and they had higher levels of Hb and Hct, suggesting that sufficient nutrient consumption could be accounted for these trends. This also suggest that the female participants might have recovered from the effects of long-COVID on Hb and Hct, but a poorly balanced diet might be the cause of their reduced oxygen carrying capacity. These micro- and macronutrients play a significant role in the synthesis of erythrocytes and the Hb molecule, as such a deficiency in them might result in low Hct and Hb (23). The results eliminate iron, folic acid, and vitamin B_12_ deficiency as the cause of low Hct and Hb in the males that might have been indicated by the results. Studies by di Filippo et al. (24); Tosato et al. (25); Cerullo et al. (26) has found that insufficient vitamin D was associated with Long COVID syndrome, while vitamin C supplementation can be used to reduce typical symptoms as vitamins are found to participate in the response of the immune system, modify oxidative stress and inflammation, thus they may alleviate persistent symptoms. This suggests that the participants of this study might not exhibit any long term effects as they might consume sufficient vitamin B_12_ and in tandem other nutrients as well.

There are a few limitations in the study. The first is the vast difference in the sample sizes of the COVID-19 non-naive and COVID-19 naïve groups. This limitation has made comparing and associating the groups problematic, and it reduces the validity of the study significantly. A low statistical power increases the risk of false negatives, while failing to detect true effects. This could create misleading conclusions of observing effects in a study, when there are none, or observing no effects whereas there are, but not detected (27). Future studies should establish an equivalent sample size for a case-control study. The second is the shortage of significant number of different ethnic groups which could have been affected by the sampling method. Convenience sampling reduces representativeness and generalizability in a study population, which leads to a lack of external validity, and increases the risks of various biases such as observer biases (28). It is recommended that future studies employ stratified or random sampling as they reduce bias and have a degree of representativeness and easily executed as compared with convenience sampling. The sample sizes were dominated by the black ethnic group, as such there were no adjustments for ethnic groups. Studies have shown that COVID-19 impacted some ethnic groups more than others, which could be due to factors such as availability or access to health care, underlying health conditions and living conditions (29, 30). A study by Athavale et al. (31) found that black and Hispanic Americans were the most impacted that other ethnic groups in America, which was substantiated in a study by Mathur et al. (32) found that some minority ethnic population in the England were higher risk of infection and were impacted the most compared to the white population. These finding could not be substantiated by the findings of this study due to the study population comprising of predominantly black participants.

The third is the FFQ assessment used to acquire dietary intake information. The questionnaire was quite lengthy and resulted in participants leaving before completing it. The recommendation is to find a way to shorten the time of assessment. It is also highly recommended that future studies that will use this tool to supplement with biochemical markers (e.g., serum iron, ferritin) to validate their dietary data. The fourth is participants who have trypanophobia, which led to some participants having missing data, and the finger prick method is less accurate than the phlebotomy method where the collected blood is analyzed in a lab. The finger prick method uses small volumes of capillary blood making it less invasive and convenient, while phlebotomy involves large amounts of venous blood which could be used for a wide range of tests (33). It is recommended that future studies employ phlebotomy as it can provide extensive hematological results. The fifth is the cross-section design of the study which limits establishing causality and recovery timelines in the participants, as such it is recommended that similar future studies must be longitudinal studies. Fifth, participants did not provide evidence for the dietary intake and of prior positive diagnosis for COVID-19 but were trusted to be honest about volunteered information. It is recommended that future studies request lab confirmed proof of diagnosis or serological testing from participants for validity.

## 5 Conclusion

The study showed no statistical significance for Hct and Hb between the two cohorts, but the study does suggest no persistent effects of COVID-19 on Hb and Hct. Diet might have had an impact on Hb and Hct in the COVID-19 non-naive male population, as it was observed that they had the highest nutrient consumption and the highest concentrations of Hct and Hb. It is crucial to conduct similar studies with a longitudinal study design and sufficient study population size. More data and robust studies are required to establish the long-term effects of COVID-19 with more valuable methods.

## Data Availability

The original contributions presented in the study are included in the article/supplementary material, further inquiries can be directed to the corresponding author.
